# Pathogenic Cav3.2 channel mutation in a child with primary generalized epilepsy

**DOI:** 10.1186/s13041-019-0509-5

**Published:** 2019-10-24

**Authors:** Ivana A. Souza, Maria A. Gandini, Fang-Xiong Zhang, Wendy G. Mitchell, Joyce Matsumoto, Jason Lerner, Tyler Mark Pierson, Gerald W. Zamponi

**Affiliations:** 1 Department of Physiology and Pharmacology, Alberta Children’s Hospital Research Institute, Hotchkiss Brain Institute, Cumming School of Medicine, University of Calgary, 3330 Hospital Dr. NW, Calgary, AB T2N 4N1 Canada; 2 Neurology Division, Children’s Hospital Los Angeles & Department of Neurology, Keck School of Medicine of University of Southern California, Los Angeles, USA; 3 Department of Pediatrics, Division of Pediatric Neurology, David Geffen School of Medicine at UCLA, Los Angeles, CA USA; 4 Department of Pediatrics, Cedars-Sinai Medical Center, Los Angeles, CA USA; 5 Department of Neurology, Cedars-Sinai Medical Center, Los Angeles, CA USA; 6 Board of Governors Regenerative Medicine Institute, Cedars-Sinai Medical Center, Los Angeles, CA USA

**Keywords:** Cav3.2, T-type, Epilepsy, Seizure, Mutation

## Abstract

Two paternally-inherited missense variants in *CACNA1H* were identified and characterized in a 6-year-old child with generalized epilepsy. Febrile and unprovoked seizures were present in this child. Both variants were expressed in *cis* or isolation using human recombinant Cav3.2 calcium channels in tsA-201 cells. Whole-cell patch-clamp recordings indicated that one variant (c.3844C > T; p.R1282W) caused a significant increase in current density consistent with a pathogenic gain-of-function phenotype; while the other *cis*-related variant (c.5294C > T; p.A1765V) had a benign profile.

## Introduction

**Fig. 1:**
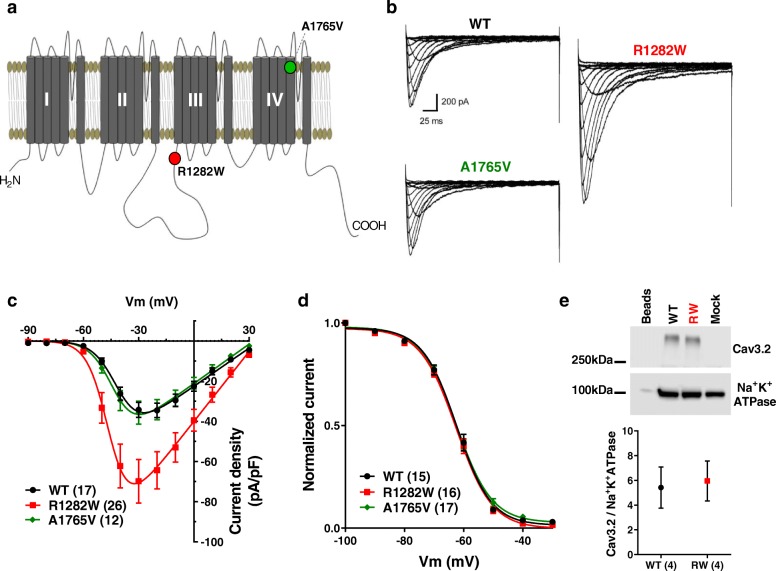
Biophysical effects of Cav3.2 mutations. **a** Schematic representation of the Cav3.2 channel showing the approximate location of the R1282W and A1765V mutations. **b** Representative Ba^2+^ current traces recorded from WT, R1282W and A1765V channels. **c** Average current densities (pA/pF) as a function of voltage for WT, R1282W and A1765V channels, showing a 2-fold increase in current densities of the R1282W mutant. **d** Steady-state inactivation curves for WT, R1282W and A1765V channels. **e** Representative western blot of surface biotinylation of WT and R1282W channels showing no difference in surface expression. Beads control represents non-biotinylated cells. Mock represents mock transfected cells. The graph shows quantified Cav3.2 integrated density normalized by Na^+^K^+^ATPase signal

*CACNA1H* encodes the α1 pore-forming subunit of the low-voltage activated (LVA) Cav3.2 T-type calcium channel. This channel is known to control neuronal excitability, plays a role in generating rebound burst-firing in thalamic reticular neurons and may contribute to neurotransmitter release at certain CNS synapses [1–3]. A number of missense mutations have been identified in *CACNA1H* in subjects with different types of generalized epilepsy that include: febrile, myoclonic, temporal lobe, and childhood and juvenile absence epilepsies [4–7]. Pathogenic variants have been identified along the entire length of the Cav3.2 channel sequence, with the most concentrated number of variants being within the intracellular region connecting the first and second transmembrane domain of the channel (see Fig. 1a). Several of these variants have been examined physiologically when expressed in heterologous systems. These studies report that a subset of these epilepsy-associated variants caused gain-of-function mutations, either through increased whole-cell current amplitude or via alterations in the inactivation properties of the channels [5, 8–11]. Although several reported variants failed to alter channel properties, it is important to remember that dysfunction may be related to the expression patterns of specific splice isoforms. For example, the Genetic Absence Epilepsy Rats from Strasbourg (GAERS) model has altered physiology when these channels contain exon 25, a splice variant transcript that is highly expressed in the thalamus [12]. Moreover, it is possible that mutations may compromise interactions with binding partners such as HCN channels [13] without affecting channel function per se. Altogether, there is considerable evidence linking Cav3.2 mutations to epilepsy in rodent models and humans.

We report two novel missense variants (p.R1282W and p.A1765V) in the *CACNA1H* gene that encodes the Cav3.2 channel in a 6-year-old girl with multifocal and primary generalized epilepsy. Electrophysiological evaluations of these two variants in *cis* and in isolation indicate that the *CACNA1H*-R1282W mutant mediates gain-of-function properties in Cav3.2 channels.

## Materials and methods

### Genetic analysis

Genomic DNA was extracted from blood from the subject and her father. Variants were identified with the Clinical Management Panel for Neurodevelopment Disorders (Courtagen, Woburn, MA).

### Molecular cloning and electrophysiology

QuikChange site-directed mutagenesis kit (Agilent Technologies) was used to insert the two variants in isolation and in *cis* into the wild-type (WT) human Cav3.2 α1 subunit gene (GenBank: NM_021098.2). The pcDNA3.1 vector was used for expression of the wild-type protein and three types of variants (p.R1282W alone, p.A1765V alone, and both together in *cis*) in tsA-201 cells.

### Cell culture, transfection, and electrophysiology

Human embryonic kidney tsA-201 cells were cultured and transiently transfected using the calcium phosphate method. Three micrograms of each Cav3.2 cDNA and 0.5 μg of eGFP were transfected and cells were grown for 72 h at 30 °C (to prevent cell overgrowth) before experiments were carried out. Whole cell patch clamp recordings were performed using an Axopatch 200B amplifier linked to a computer with pCLAMP 9.2 software. The current-voltage relationships were acquired and fitted with a Boltzmann equation as described previously [14].

Whole cell patch clamp recordings were performed at room temperature (22–24 °C) using an Axopatch 200B amplifier linked to a computer with pCLAMP 9.2 software. The external solution contained (in mM): 10 BaCl_2_, 125 CsCl, 1 MgCl_2_, 10 HEPES and 10 Glucose, pH adjusted to 7.4 with CsOH. The internal pipette solution contained (in mM): 130 CsCl, 2.5 MgCl_2_, 10 HEPES, 5 EGTA, 3 ATP 0.5 GTP, pH 7.4. The current-voltage relationships were acquired and fitted with a Boltzmann equation as described previously [14]. Steady-state inactivation was acquired by applying 1 s conditioning pulses from − 100 to − 30 mV in 10 mV increments followed by a 25 ms test pulse to − 20 mV. Currents were normalized to the maximum current and fitted with a Boltzmann equation. Recovery from inactivation was evaluated by applying two test pulses (P1 and P2) to − 20 mV separated by a varying interval ranging from 10 ms to 2 s. P2/P1 was plotted as a function of time. Data were analyzed using Clampfit 10.3 software (Molecular Devices) and fit using GraphPad Prism 6. Averaged data are plotted as mean ± SE and statistical analysis was performed using Student’s *t* tests, where *p* ≤ 0.05 was considered significant.

## Results

### Subject

The subject is a six-year-old female with primary generalized epilepsy and a paternal history of childhood absence epilepsy that resolved “within a few years”. The child was born at 33.5 weeks gestation via emergent cesarean section due to maternal pre-eclampsia and had an uncomplicated three-week NICU stay prior to discharge. Her development was normal, with her first words at 10 months and ambulation at 14 months. Her physical and neurological examinations have consistently been within normal limits.

Her first convulsive seizure was at 14 months in the setting of a febrile illness. She had two more febrile convulsive episodes within the next month that were noted to start with right-sided twitching. She had ten additional convulsions in the setting of fever during the following year, most of which were prolonged and required abortive benzodiazepine treatment. She had her first unprovoked generalized tonic-clonic seizure at 2 years of age. The child was then started on Leviteracetam and did well for some time, but developed aggressive behavior and was switched to lamotrigine. She has had no further seizures since starting anti-seizure medications, except in the setting of febrile illnesses.

### Neurodiagnostic testing

Electroencephalogram (EEG) and brain MRI were normal after her first seizures at 17 months. A subsequent EEG after her first unprovoked seizure at 2 years had bilateral multi-focal discharges (right greater than left) arising from the frontal central and anterior temporal regions, along with generalized spike and multispike and slow wave discharges of 3–4 Hz with bifrontal predominance. At 6 years, another EEG showed frequent generalized 2–2.5 Hz spike and wave discharges with bifrontal predominance lasting up to 10 s. This EEG was also notable for one episode of behavioral arrest lasting 5 s during hyperventilation that was associated with a burst of 2.5 Hz generalized spike and wave discharges (consistent with an absence seizure).

### Genetic testing

Genetic sequencing revealed two paternally-inherited missense variants in the *CACNA1H* gene (c.3844C > T, p.R1282W and c.5294C > T, p.A1765V) in the same allele. The variant A1765V, located in the transmembrane region S5 of domain IV (Fig. 1a), had been previously observed in 4 out of 24,702 individuals in the ExAC database (http://exac.broadinstitute.org); while the R1282W variant was located in the cytoplasmic II-III loop (Fig. 1a) and was not found within this database.

### Electrophysiology

Whole-cell patch clamp recordings of Cav3.2 channels containing the variants R1282W and A1765V were compared to wild-type channels (WT). Representative current traces for WT, R1282W and A1765V channels are shown in Fig. 1b. The peak current density recorded from cells expressing R1282W was significantly increased (*p* < 0.05, One Way ANOVA followed by Tukey’s multiple comparisons test) when compared to WT (Fig. 1c, Table 1). This effect was not due to a change in cell surface expression of the channel as determined by surface biotinylation experiments (Fig. 1e). Neither of the mutations caused a change in steady-state inactivation and recovery from inactivation properties of the channel compared to WT, nor did they affect the half-activation potential (Fig. 1d, Table 1).

**Table 1:** Summary of electrophysiological parameters of wild-type (WT), R1282W and A1765V channels expressed in tsA-201 cells

	Peak current density (pA/pF)	*G*max (nS)	V_1/2_ act (mV)	V_1/2_ inac (mV)	τrecov (ms)
WT	−34.39 ± 3.7 (17)	0.6 ± 0.06 (17)	−40.94 ± 0.9 (17)	−62.1 ± 0.9 (15)	239.4 ± 28.5 (14)
R1282W	−69.8 ± 10.8 (26)*	1.1 ± 0.15 (26)*	−43.5 ± 1.0 (26)	−62.2 ± 0.8 (16)	287.1 ± 26.9 (9)
A1765V	−35.7 ± 5.6 (12)	0.6 ± 0.09 (12)	− 41.9 ± 0.9 (12)	−62.3 ± 0.6 (17)	259.3 ± 25.4 (11)

Data are represented as mean ± SEM. Numbers in parentheses represent number of cells analyzed. *p < 0.05, One Way ANOVA followed by Tukey’s multiple comparison test

**Fig. 2:**
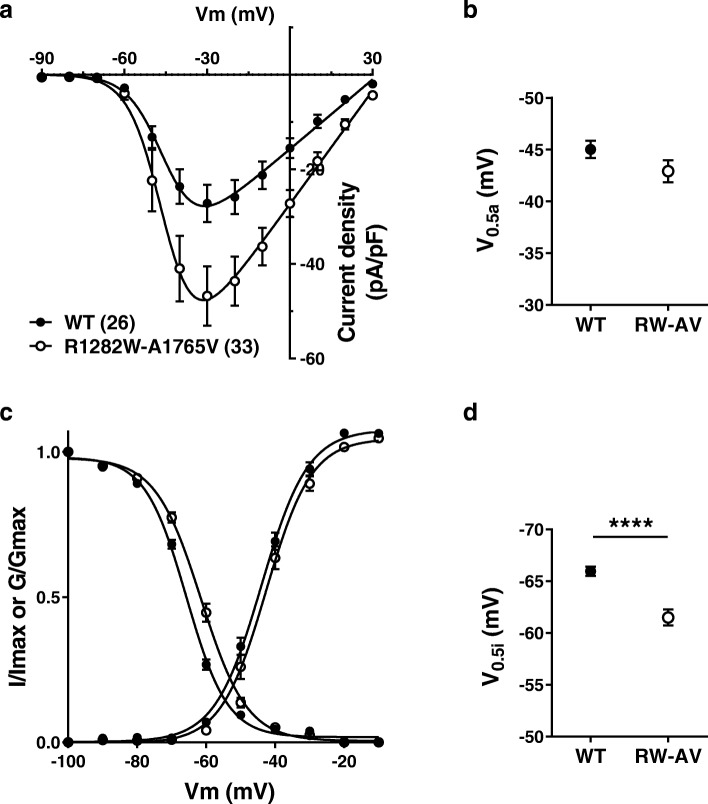
Biophysical analysis of variants R1282W and A1765V in *cis*. **a** Average current densities (pA/pF) as a function of voltage for WT and R1282W-A1765V channels, showing an increase in current density for R1282W-A1765V. Numbers in parentheses reflect numbesr of cells. **b** Mean half-voltage of activation values for R1282W-A1765V compared to WT channels. **c** Steady-state of inactivation curves for WT and R1282W-A1765V channels. The steady state activation curves extracted from the current-voltage relations in panel (**a**) are superimposed to highlight the window currents. **d** Mean half-voltage of inactivation values for R1282W-A1765V compared to WT channels. ****p < 0.0001, Student’s t-test

The transmembrane A1765V mutant in isolation was similar to WT channels in all of the parameters tested (Fig. 1b-d, Table 1). We also examined possible synergistic effects of the two mutations when placed within the same allele. These experiments showed that A1765V produced no additive effects on current density and activation kinetics to those observed with R1282W alone (Fig. 2a-b); however, the mean half-inactivation voltage was slightly shifted to more a positive potential (*p* < 0.0001, Student’s t-test) (Fig. 2c-d). This in turn results in an increase in the size of the normalized window current by 15%, overall consistent with a gain of function beyond the increase in whole cell current density. The mechanistic underpinnings of this additive effect on inactivation are unclear.

## Discussion

*CACNA1H* pathogenic variants have been associated with multiple disorders, including aldosteronism, autism, amyotrophic lateral sclerosis, but is predominantly associated with epilepsy [15–17]. Here, we characterized the functional effects of two missense variants identified in the *CACNA1H* gene from a patient with epilepsy. These variants were introduced into the Cav3.2 channel isoform that contains the exon 25, a subtype that is highly expressed in the thalamus [12]. The A1765V mutation alone had no effect on any of the parameters tested and was predicted to be benign by SIFT software analysis (http://sift.bii.a-star.edu.sg). These data and its frequency in the population indicate this may indeed likely be a benign variant. It is important to note that some point mutations found in calcium channels can also behave differently when introduced into different isoforms of the channel [12], when channels are expressed in a different cellular milieu [18], or recorded in different external solutions [14]. Moreover, it has been shown that T-type channels gate much faster at physiological temperatures [19], and it is possible that mutations may affect channel behavior differently at 37 °C compared to room temperature where we conducted our analysis. In contrast, the R1282W variant was a novel variant that SIFT analysis predicted to potentially alter protein function. When compared to WT Cav3.2 channels, the R1282W-variant was shown to cause a significant 2-fold increase in whole cell current density (Fig. 1b-c, Table 1), consistent with a gain-of-function mutation. Given that cell surface expression was unaltered for this variant, these data indicate that the mutation may have either increased the single channel conductance or the maximum open probability at the plateau of the activation curve.

T-type channel dysfunction fits well with a pathogenic model of this disorder as they are important for the thalamocortical network, where they contribute to generation of bursts of action potentials and network oscillations [2]. Therefore, an increase in T-type channel activity can shift the balance between excitatory and inhibitory neurotransmission and increase seizure susceptibility. This is also supported by genetic rodent models of epilepsy, such as GAERS and the Wistar Albino Glaxo Rats from Rijswijk (WAG/Rij), which exhibit an increase in T-type channel function in nRT neurons [20, 21]. However, although certain mutations in Cav3.2 channels have been previously associated with an increased incidence of febrile seizures [5], this may not necessarily involve a dysfunction of the thalamocortical network. Importantly, T-type calcium channels are prominently expressed in other brain structures that are linked to febrile seizure disorders, including the hippocampus, and have been shown to play an important role in generating seizure activity in this brain region [22–26].

The impact of variants identified in patients with different disorders can be very difficult to predict without the functional analysis of the affected proteins. The increase in availability of high through-put sequencing data have provided numerous examples where functional analysis is important to confirm pathogenicity in rare disorders in which some potentially pathogenic variants are found to be quite common in the general unaffected population and therefore likely benign [27]. This becomes an even greater challenge when studying complex diseases such as epilepsy, which may have polygenic and/or multifactorial origins. Many variants identified in the *CACNA1H* gene have shown little physiological alterations when tested in vitro and are not considered sufficiently pathogenic to cause epilepsies on their own [5, 9]; however, they are believed to act in combination with variants in other susceptibility genes and/or environmental factors to raise the level of neuronal excitability and generate seizures [28]. In addition, it is possible that even small changes in electrophysiological properties of Cav3.2 channels may affect downstream signaling processes such as calcium-dependent gene transcription, which in turn may lead to the dysregulation of pathways that culminate in increased seizure susceptibility. In summary, a functional analysis of these two *cis*-linked variants revealed that only one of them caused a novel pathogenic gain-of-function (R1282W) mutation in *CACNA1H*, while the other variant seems likely benign.

## Data Availability

The data used in our study are available from the authors on reasonable request.

## Abbreviations

EEG: Electroencephalogram
NICU: Neonatal intensive care unit
nRT: nucleus reticularis
